# Altered localization and functionality of TAR DNA Binding Protein 43 (TDP-43) in niemann- pick disease type C

**DOI:** 10.1186/s40478-016-0325-4

**Published:** 2016-05-18

**Authors:** A. Dardis, S. Zampieri, S. Canterini, K. L. Newell, C. Stuani, J. R. Murrell, B. Ghetti, M. T. Fiorenza, B. Bembi, E. Buratti

**Affiliations:** Regional Coordinator Centre for Rare Diseases, University Hospital Santa Maria della Misericordia, Udine. P.le Santa Maria della Misericordia 15, 33100 Udine, Italy; Department of Psychology, Section of Neuroscience and “Daniel Bovet” Neurobiology Research Center, Università “La Sapienza”, Roma, Italy; Department of Pathology & Laboratory Medicine, University of Kansas School of Medicine, Kansas City, Kansas; International Centre for Genetic Engineering and Biotechnology, Area Science Park, 34194 Trieste, Italy; Department of Pathology & Laboratory Medicine, Indiana University School of Medicine, Indianapolis, Indiana

**Keywords:** Niemann Pick C, TDP-43, Lysosomal diseases, NPC1

## Abstract

Niemann-Pick type C (NPC) disease is a lysosomal storage disorder characterized by the occurrence of visceral and neurological symptoms. At present, the molecular mechanisms causing neurodegeneration in this disease are unknown. Here we report the altered expression and/or mislocalization of the TAR-DNA binding protein 43 (TDP-43) in both NPC mouse and in a human neuronal model of the disease. We also report the neuropathologic study of a NPC patient’s brain, showing that while TDP-43 is below immunohistochemical detection in nuclei of cerebellar Purkinje cells, it has a predominant localization in the cytoplasm of these cells. From a functional point of view, the TDP-43 mislocalization, that occurs in a human experimental neuronal model system, is associated with specific alterations in TDP-43 controlled genes. Most interestingly, treatment with N-Acetyl-cysteine (NAC) or beta-cyclodextrin (CD) can partially restore TDP-43 nuclear localization. Taken together, the results of these studies extend the role of TDP-43 beyond the Amyotrophic lateral sclerosis (ALS)/frontotemporal dementia (FTD)/Alzheimer disease (AD) spectrum. These findings may open novel research/therapeutic avenues for a better understanding of both NPC disease and the TDP-43 proteinopathy disease mechanism.

**Electronic supplementary material:**

The online version of this article (doi:10.1186/s40478-016-0325-4) contains supplementary material, which is available to authorized users.

## Introduction

Niemann-Pick disease, type C [NPC-MIM 257220; MIM607625] is an autosomal recessive lysosomal storage disorder due to mutations in *NPC1* (95 % of patients) or *NPC2* genes, encoding two proteins involved in the intracellular trafficking of cholesterol and other lipids. The deficiency of either protein leads to the accumulation of endocytosed unesterified cholesterol, gangliosides, and other lipids within the lysosome/late endosome compartment [1].

The clinical presentation of the disease is variable, and the age at onset ranges from the perinatal period to adulthood. The disease is typically characterized by visceral and neurological symptoms. Apart from a small group of patients presenting with a severe perinatal form, leading to death within the first 6 months of life due to liver or respiratory failure, most patients develop a progressive neurological disease. Indeed, NPC has been classically classified on the basis of the age at onset of the neurological symptoms, irrespective of the age of first symptoms [1].

Neuropathological features include meganeurite formation, extensive growth of ectopic dendrites, formation of neurofibrillary tangles, neuroinflammation and neuroaxonal dystrophy [2]. In advanced stages of the disease, neuronal loss is prominent, affecting particularly cerebellar Purkinje cells [3]. At present, however, the molecular events linking lysosomal storage and cellular damage are not well understood.

Recently, it has been suggested that synergistic mechanisms, between pathological proteins involved in neurodegeneration (beta-amyloid, tau, alpha-synuclein, TDP-43), may help explain copathologies and individual diversity that are often detected in neurodegenerative conditions [4]. In particular, TAR-DNA binding protein 43 (TDP-43), a member of the heterogeneous nuclear ribonucleoproteins (hnRNPs) family [5], has emerged as a new player in the field of neurodegenerative diseases [6]. Like many hnRNPs, the major functional role of this protein in cellular metabolism concerns the regulation of many steps in RNA processing [7]. In 2006, aberrantly phosphorylated and ubiquitinated TDP-43 was identified as a main component of the cytoplasmic neuronal inclusions in patients affected by amyotrophic lateral sclerosis (ALS) and frontotemporal lobar degeneration (FTLD) [8]. More recently, pathological TDP-43 inclusions have also been found in other neurodegenerative disorders such as Alzheimer’s, Parkinson’s, Huntington’s and Alexander diseases and Perry Syndrome [9–12].

At the pathological level, it is now very clear that both gain of toxic function of cytoplasmic aggregates and loss of TDP-43 function in the nucleus may contribute to disease pathogenesis [13, 14]. What is still the focus of intensive research is the characterization of the molecular pathways that are linked with aberrant TDP-43 aggregation. However, several recent lines of evidence indicate that the autophagic and proteasome systems play a major role in the clearance and intracellular localization of TDP-43 [15–17].

Considering this evidence, it is reasonable to hypothesize that lysosomal dysfunction might be linked with TDP-43 proteinopathy. Indeed, it has been shown that GGGGCC intronic expansions of the *C9orf72* gene, encoding a protein involved in the regulation of the endosomal trafficking [18], are mostly associated with the presence of TDP-43 pathology in disease-relevant brain regions [19]. In addition, evidence of lysosomal impairment has been recently found in brains from patients affected by frontotemporal lobar degeneration (FTLD) with accumulation of TDP-43 due to heterozygous loss-of-function mutations in the progranulin (*GRN*) gene. Interestingly, a *GRN* loss-of-function, caused by a homozygous 4-bp deletion in the *GRN* gene has been associated with NCL11, a form of neuronal ceroid lipofuscinosis, in which the lysosomal storage disorder coexists with the accumulation of phosphorylated TDP-43 in neurons. Thus, lysosomal storage disorders and FTLD may share common features [20].

Defective autophagy, characterized by increased autophagosomal formation and slower turnover of autophagosomes due to impaired lysosomal proteolysis, represents a main feature of NPC pathology [21–24].

Based on this evidence, we hypothesized that TDP-43 localization/expression might be altered in NPC cells and that these alterations may contribute to disease pathogenesis. Therefore, we analyzed the expression and intracellular localization of TDP-43 both in mice and human models of NPC disease.

## Materials and methods

### Mice

Npc1nih/nih mice maintained on BALB/cJ background were obtained from heterozygous mating and genotypes were identified at weaning by PCR on tail DNA as previously described [25]. All mice were maintained in our animal facility in accordance with the institutional (Università la Sapienza) guidelines for the care and use of laboratory animals. Experimental protocols and related procedures were approved by the Italian Ministry of Public Health.

### Human samples

Skin fibroblasts were obtained from 4 healthy donors and 4 patients affected by NPC disease diagnosed at the Regional Coordinator Centre for Rare Diseases by filipin staining of cultured fibroblasts and genetic testing of *NPC1* and *NPC2* as previously described [26]. Patient’s genotypes are reported in Table 1. The presence of mutations in cultured cells was also verified.

**Table 1:** Genotypes of patients included in the study

Patient	Allele 1	Allele 2
NPC-1	c.3182 T > C (p. I1061T)	c.3182 T > C (p.I1061T)
NPC-2	c.3182 T > C (p.I1061T)	c.3182 T > C (p.I1061T)
NPC-3	c.2291C > T (p.A764V)	c.2819C > T (p.S940L)
NPC-4	c.2800C > T (p.R934X)	c.3235 T > C (p.F1079L)

The lysosomal accumulation of unesterified cholesterol was demonstrated by filipin staining. All cells presented a classical biochemical phenotype characterized by massive lysosomal accumulation of unesterified cholesterol

Brain tissue was obtained from a 61 year-old man who died after a 30-year history of a clinically undiagnosed, familial, neurological disorder. Molecular genetic testing of DNA extracted from frozen brain autopsy tissue [27] revealed two pathogenic mutations in the *NPC1* gene, in intron 14 (IVS14-2A > G) and exon 18 [c.2621A > T (D874)].

All procedures followed were in accordance with the ethical standards of the responsible committee on human experimentation (institutional and national) and with the 1964 Helsinki declaration and its later amendments or comparable ethical standards.

Informed, written consent was obtained from the patients (who underwent skin biopsies) included in this report.

### Cell culture

Fibroblasts were cultured and maintained in Dulbecco’s modified Eagle’s medium (DMEM) (Gibco, Paisley, UK) containing 10 % fetal calf serum (FCS) and penicillin/streptomycin, in a humidified atmosphere containing 5 % CO2 at 37 °C.

Multipotent adult stem cells were obtained from fibroblasts at early passages (SKIN-MASCs) from 4 healthy donors and 4 patients affected by NPC disease, as previously described [28]. The surface immunophenotype was determined using the following primary conjugated antibodies: CD13, CD49a, CD49b, CD49d, CD90, CD73, CD44, CD45, human leukocyte antigen-D related (HLA-DR), CD34, and CD271 (BD Biosciences, Franklin Lakes, NJ, USA); CD105 and kinase insert domain receptor (KDR; Serotec, Oxford, United Kingdom); and CD133 (Miltenyi Biotec, Bergisch Gladbach, Germany). The percentage of cells expressing all the antigens was determined by fluorescence-activated cell sorting (FACS) analysis (CyAn; Beckman Coulter, Brea, CA, USA). Properly conjugated isotype-matched antibodies were used as negative controls.

### Neuronal differentiation

SKIN-MASCs obtained after 3 passages in selective medium, were seeded at a density of 8000 cells/cm^2^ into plates (Corning) or on coverslips. The differentiation protocol was previously described by Bergamin et al. [28]. Cells were plated in medium containing DMEM-HG with 10 % FBS (N1 medium). After 24 h the DMEM-HG was replaced with fresh medium supplemented with 1 % of B27 (Invitrogen), 10 ng/ml EGF (Peprotech) and 20 ng/ml bFGF (Peprotech) (N2 medium) for 5 days. Thereafter, cells were incubated for 72 h in DMEM-HG supplemented with 5 μg/ml insulin, 200 μM of indomethacin and 0.5 mM IBMX (all from Sigma-Aldrich) without FBS (N3 medium). The actual differentiation was determined by analyzing the expression of the neuron specific markers, NeuN and tubulin b3.

### Treatments

NPC neuronally differentiated cells were treated with vehicle or either 100 μΜ M N-acetyl cysteine (NAC, Sigma) or 400–800 μM 2-hydroxypropyl-b-cyclodextrin (CD, Sigma Aldrich, Milan, Italy) for 24 h.

### Histology, immunohistochemistry and immunofluorescence

*Npc1*^*+/+*^ and *Npc1*^*−/−*^ mice of postnatal days 11, 28 and 75 (PN11–75; 3 mice/genotype/age) were deeply anesthetized and then transcardially perfused with 4 % paraformaldehyde (PFA) in PBS. Brains were removed, and processed for immunohistochemical studies, according to a previously published protocol [29], TDP-43 immunostaining was performed using a rabbit polyclonal anti-TDP-43 antibody (Proteintech, 1:200 dilution in PBS), and then using the rabbit Vectastain Elite ABC Kit (Vector Laboratories Inc., Burlingame, CA, USA) and peroxidase substrate Vector VIP (Vector Laboratories Inc), according to manufacturer’s instructions.

For immunofluorescence assays cells were fixed for 15 min in 4 % (w/v) paraformaldehyde in PBS and then permeabilized in 0.3 % Triton X-100 in PBS for 5 min on wet ice. After blocking with 2 % BSA in PBS, cells were incubated overnight at 4 °C with the primary antibodies raised against NeuN (Millipore), tubulin b3 (Covance, Inc., Princeton, NJ, USA), TDP-43 (Proteintech) Phospho-TDP-43 (Ser409/410 Sigma Aldritch), TIA-1 (Santa Cruz), Lamp-1 (Santa Cruz) and p62 (MBL). Cells were then washed and incubated with Alexa Fluor 555 or 488 labeled secondary antibody for 1 h at 37 °C. Cell nuclei were stained by Vectashield Mountain Medium with DAPI (Vector Laboratories, Inc., Burlingame, CA, USA).

Images were obtained with a live cell imaging dedicated system consisting of a Leica DMI 6000B microscope connected to a Leica DFC350FX camera (Leica Microsystems, Wetzlar, Hessen, Germany).

### Preparation of nuclear and cytoplasmic fractions

Nuclear and cytoplasmic fractions were prepared as previously described [30], with few modifications. Briefly, PN11-75 cerebella (3 mice/genotype/age) were homogenized using a glass Dounce homogenizer in 10 mM HEPES, pH 7.9, 10 mM KCl, 0.1 mM EDTA, 0.1 mM EGTA, 1 mM DTT and protease/phosphatase inhibitor (Roche Diagnostics, Indianapolis, IN, USA). Homogenates were incubated with 0, 5 % NP-40 for 10 min on ice and centrifuged at 600 g for 5 min at 4 C. The supernatant (total cytoplasmic fraction) was diluted with Laemmli buffer and the pellet (total nuclear fraction) was resuspended in 20 mM HEPES, pH 7.9, 420 mM NaCl, 1 mM EDTA, 1 mM EGTA, 1 mM dithiothreitol and protease/phosphatase inhibitors (Roche Diagnostics), extracted for 15 min on ice and cleared by centrifugation at 10 000 g for 10 min. The supernatant was finally diluted in Laemmli buffer. Protein concentration was routinely determined using the DC Protein Assay (Bio-Rad Laboratories, Hercules, CA, USA).

### Western blot analysis

Equal amounts per lane of cytoplasmic and nuclear protein fractions were loaded on a 4-20 % gradient Mini-Protean TGX pre-cast gel for electrophoresis (Bio-Rad Laboratories) while total extracts were electrophoresed on 10 % SDS–PAGE. Fractionated proteins were transferred to poly (vinylidene difluoride) membranes (Roche Diagnostics) or nitrocellulose membrane (Biorad, Hercules, CA, USA), respectively. After blocking, membranes were incubated overnight at 4 °C with the primary antibody anti-TDP-43 (Protein Tech), then washed, incubated with the appropriate secondary antibody for 1 h at RT and developed with SuperSignal West Dura reagents (Thermo Scientific/Pierce, Rockford, IL, USA). Sp1 and β-III tubulin were used to assess the purity of nuclear and cytoplasmic fractions, respectively, and as reference for TDP-43 expression quantification. Total expression of TDP-43 was normalized by actin levels. Blots were quantified by using a Gel Doc 2000 videodensitometer (Biorad, Hercules, CA, USA).

### RNA extraction, reverse transcription and quantitative real-time PCR

Total RNA was extracted from neuronally differentiated cells using the RNeasy Mini RNA extraction kit (Qiagen) according to the manufacturer’s protocols. First strand cDNA synthesis was performed with 1ug RNA using random hexanucleotides (Invitrogen) and SuperScript III reverse transcriptase (Invitrogen). Quantitative RT-PCR was carried out in 15 μl reaction volume containing 1-3ul of obtained cDNA, SsoAdvanced Universal SYBR Green Supermix (BioRad) and gene-specific sense and anti-sense primers. HPRT was used as an internal control for normalization. Primer sequences are listed in Additional file 1: Table S1.

Quantitative RT-PCR was performed in a Roche 480 LightCycler Real-Time PCR System, following the manufacturer’s instructions. LightCycler 480 Basic software (Roche) utilized the second derivative maximum method to identify the crossing point (Cp).

### Neuropathologic studies

Neuropathologic studies of brain and spinal cord were carried out in order to characterize NPC in human brain tissues. Samples of fresh brain tissue were frozen and stored at −80° C. The remainder of the brain and spinal cord was fixed in 10 % buffered formalin. Fixed tissue sections were obtained from multiple brain and spinal cord regions and processed for histology and immunohistochemical studies, according to a previously published protocol [31]. TDP43 immunostaining was simultaneously performed on the cerebellum of four control subjects.

### Statistical analysis

Statistical significance was determined by Student’s t-test, using the SPS software. *p* < 0.05 was considered as statistically significant.

## Results

### Analysis of TDP-43 in NPC mice

**Fig. 1:**
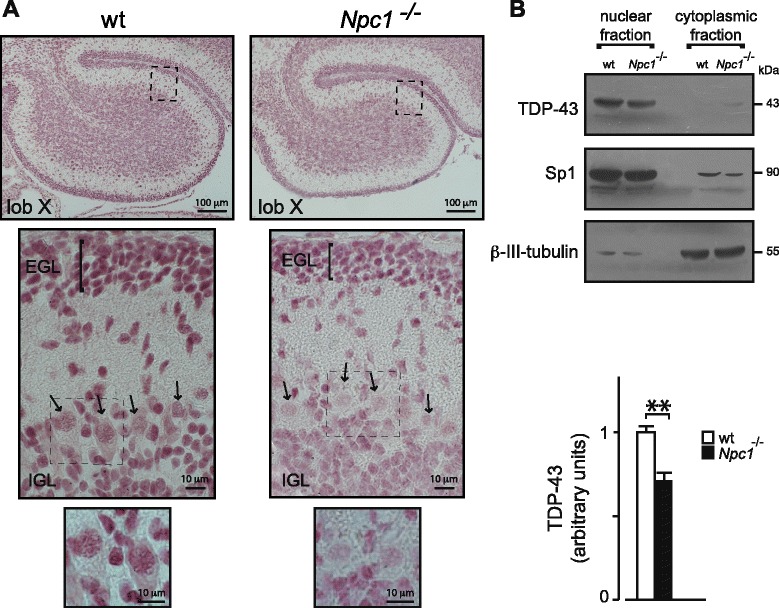
TDP-43 immunohistochemistry of cerebellum sections of wt and *Npc1* ^*−/−*^ PN11 mice (**a**). At higher magnification (bottom panels), immunohistochemistry revealed a decrease of TDP-43 nuclear expression in GNs of both external granule layer (EGL) and internal granule layer (IGL), and in Purkinje cells (*arrows*) of *Npc1* ^*−/−*^ mice compared to wt. **b** Western blot analysis of TDP-43 protein levels in nuclear and cytoplasmic cerebellar fractions, identified by Sp1 and β-III-tubulin, respectively. Histograms indicate nuclear TDP-43 abundance (mean ± SEM), determined by densitometry of protein bands obtained in at least 3 independent experiments (having at least three replicates for each data point) taking Sp1 as internal reference. ** indicates *P* < 0.01 in Student’s t-test

The expression and subcellular localization of TDP-43 in the cerebellum of *wild-type* and *Npc1*^*−/−*^ mice during maturation and at adult age were investigated by immunohistochemistry and western blot, respectively (Fig. 1a, b). The expression of TDP-43 appeared significantly reduced in the cerebellum of *Npc1*^*−/−*^ mice as early as postnatal (PN) day 11, when neuropathological features of the disease had not yet appeared. Because the development of cerebellar cortex is still in progress at this age, three distinct granule neurons (GN) populations, consisting of proliferating, postmitotic and migrating GNs can be easily identified. Proliferating GNs localize in the outer part of the external granular layer (oEGL) whereas postmitotic GNs stop in the inner part of the EGL (iEGL) before migrating inward through the molecular layer (ML) to reach the internal granular layer (IGL), thus representing the migrating GNs. Despite a reduction of TDP-43 expression level in *Npc1*^*−/−*^ mice, EGL-residing GNs of both *wild-type* and *Npc1*^*−/−*^ mice expressed higher levels of this protein compared to IGL-residing GNs. This may indicate a burst of TDP-43 expression in concomitance with GN exit from the cell cycle. A significant reduction of TDP-43 expression in *Npc1*^*−/−*^ compared to *wild-type* mice was also observed in Purkinje cells (PCs) (Fig. 1a). In both GNs and PCs TDP-43 prominently localized to the nucleus. The reduced expression of TDP-43 in the cerebellum of PN11 *Npc1*^*−/−*^ mice was also assessed by western blot analysis, observing a significant reduction of the protein level (25–30 %) and confirming the nuclear localization of TDP-43 (Fig1B).

**Fig. 2:**
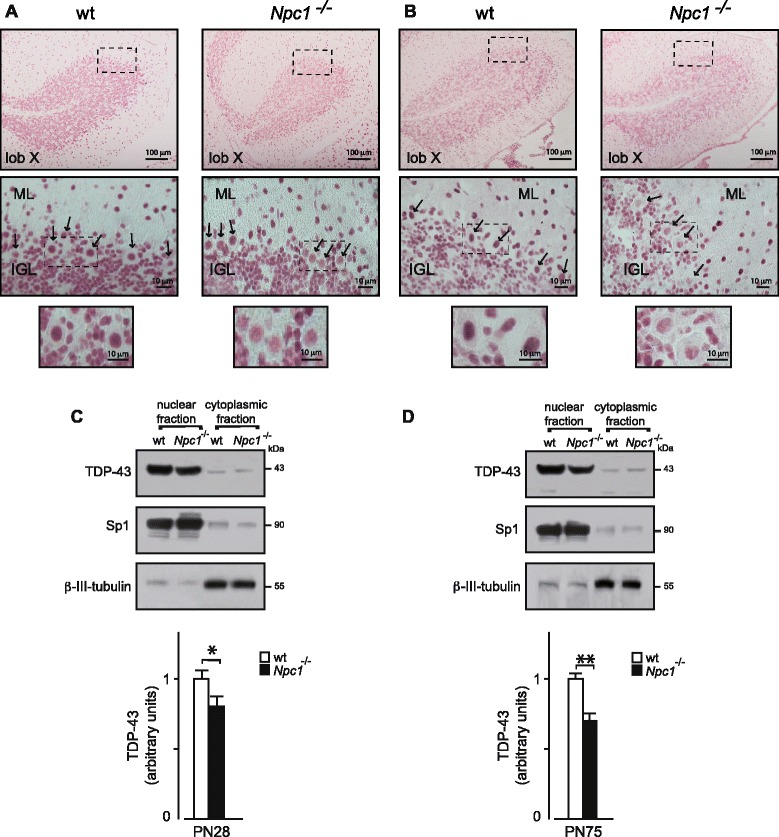
TDP-43 immunohistochemistry of cerebellum sections of wt and *Npc1* ^*−/−*^ PN28 (**a**) and PN75 (**b**) mice. At higher magnification (*bottom panels*), immunohistochemistry revealed a decrease of TDP-43 nuclear expression in Purkinje cells (*arrows*) and GNs of *Npc1* ^*−/−*^ compared to wt mice, which is particularly pronounced in PN75 mice. ML: molecular layer; IGL: inner granule layer. (**c** and **d**) Western blot analysis of TDP-43 protein levels in nuclear and cytoplasmic cerebellar fractions, identified by Sp1 and β-III-tubulin, respectively. Histograms indicate nuclear TDP-43 abundance (mean ± SEM), determined by densitometry of protein bands obtained in at least 3 independent experiments (having at least three replicates for each data point) taking the Sp1 as internal reference. * indicates *P* < 0.05 and ** indicates *P* < 0.01 in Student’s t-test

A similar TDP-43 expression level reduction was observed between *wild-type* and *Npc1*^*−/−*^ PN28 mice (Fig. 2a and c). At this stage, TDP-43 appeared to be particularly abundant in PC nuclei of *wild-type* mice, whereas both PCs and GNs of *Npc1*^*−/−*^ mice displayed a reduced level of TDP-43. A similar TDP-43 expression pattern was displayed by additional neuronal cell types, including basket and stellate cells that at this age can be easily detected within the ML.

Finally, in adult PN75 *Npc1*^*−/−*^ mouse cerebella the expression of TDP-43 was minimally detected in any of the neuronal cell types, including PCs, GNs, basket and stellate cells, whereas *wild-type* mouse cerebella displayed a TDP-43 expression pattern that matched that observed in PN28 mice (Fig. 2b and d).

These results indicate that a progressive reduction of TDP-43 expression occurs in the cerebellum of *Npc1*^*−/−*^ with increasing age and is present only in the disease mice.

### Analysis of TDP-43 in a human neuronal model of NPC

To extend these observations to the human disease, we then analyzed the expression of TDP-43 mRNA and protein, both in cultured fibroblasts and in a human neuronal model of NPC. This model was obtained through the differentiation of human multipoint adult stem cells isolated from the skin (hSKIN-MASCs) of patients affected by NPC. As we have previously shown, this neuronal model has several key features of NPC and is therefore valuable for studying the effects of NPC dysfunction at the cellular level [28].

**Fig. 3:**
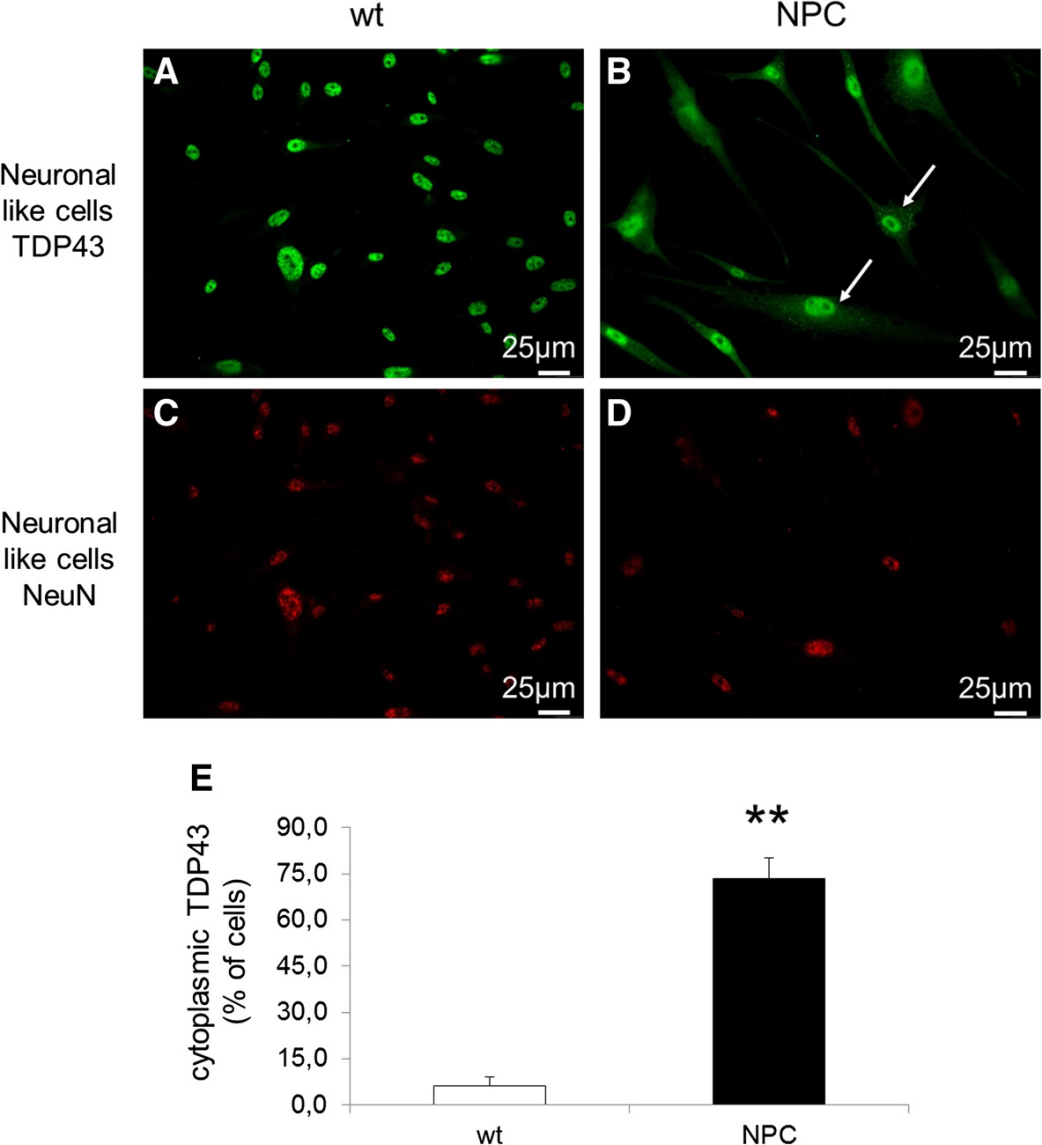
Intracellular localization of TDP-43 (labeled in green) in neuron-like cells obtained from wt (**a**) and NPC patients (**b**). Immunofluorescence showed diffuse cytosolic distribution of TDP-43 in NPC neuronal-like cells (indicated by arrows) while in wt cells TDP-43 was confined to the nucleus. The actual neuronal differentiation of cells was confirmed by immunofluorescence using the neuronal marker NeuN (labeled in *red*) (**c**-**d**). **e** Quantitative evaluation of the percentage of neuron like cells displaying cytoplasmic localization of TDP-43 in wt (*n* = 4) and NPC patients (*n* = 4). At least 400 cells have been counted for each cell line. Data are presented as mean ± SD of 3 independent experiments. **p < 0.01 in Student’s t test

**Fig. 4:**
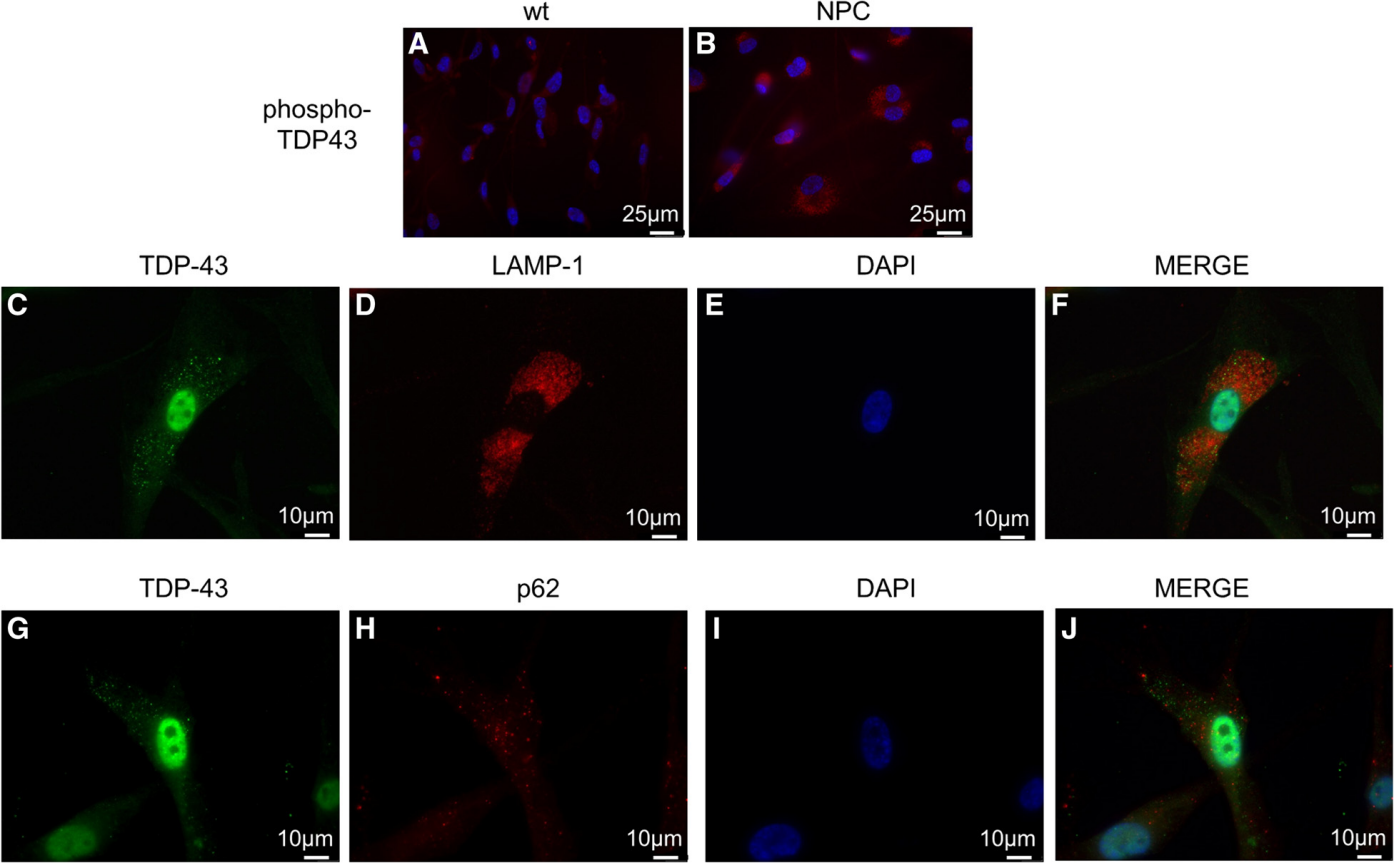
Immunofluorescence of phospho-TDP-43 (labeled in *red*) in wt (**a**) and NPC (**b**) neuron-like cells. TDP-43 immunostaining using a phospho-specific anti-TDP43 antibody showed the cytosolic accumulation of phosphor-TDP-43 in NPC neuronal-like cells. **c**-**f** Co-Immunofluorescence of TDP-43 (labeled in *green*) and the lysosomal marker LAMP-1 (labeled in *red*). **g**-**j** Co-Immunofluorescence of TDP-43 (labeled in *green*) and the autophagic marker p62 (labeled in *red*). Immunostaining showed that neither LAMP-1 or p62 co-localized with TDP-43. Nuclei were stained with DAPI (*blue*)

The levels of both mRNA and protein TDP-43 expression in NPC cells were not significantly different from the levels detected in cells derived from normal controls (Additional file 2: Figure S1). However, when the intracellular distribution of TDP-43 was analyzed by immunofluorescence, a completely different intracellular localization of this protein was observed in NPC compared to control cells. As shown in Fig. 3a, b and e, in healthy control cells TDP-43 was confined within the cell nuclei as previously reported by many studies, while in most NPC-derived cells (about 73 %) a significant amount of TDP-43 protein was localized in the cytosol, where it showed a diffuse distribution. As expected, differentiated cells stained positively for NeuN, a marker of mature neurons (Fig. 3c and d). Furthermore, immunostaining with an antibody against the phosphorylated form of TDP-43 showed that in NPC differentiated cells TDP-43 mislocalization is associated with hyperphosphorylation (Fig. 4a and b, upper panels).

Then, we analyzed whether the observed cytoplasmic accumulation of TDP-43 in NPC cells is a consequence of the autophagic build up caused by defective autophagosomal efflux. However, as shown in Fig. 4 (lower panels) TDP-43 did not colocalize with lysosomal (LAMP-1, Fig. 4c-f) or autophagosomal (p62, Fig. 4g-j) markers in NPC derived cells.

**Fig. 5:**
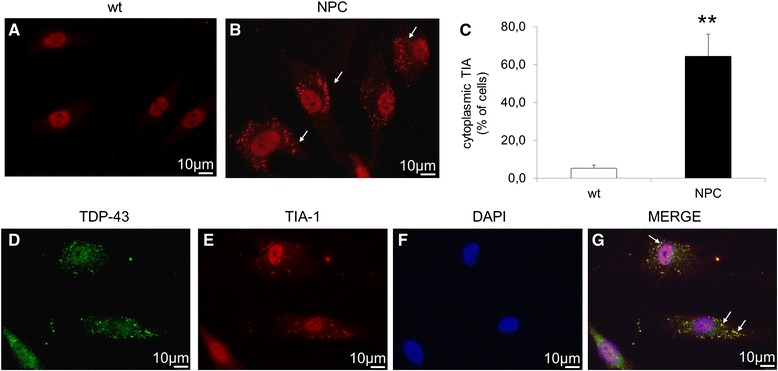
**a** Immunofluorescence of the stress granule marker TIA-1 in wt (**a**) and NPC (**b**) neuron-like cells. Arrows indicate the cytosolic localization of stress granules in NPC cells. **c** Quantitative evaluation of the percentage of neuron like cells displaying cytoplasmic localization of TIA- in wt (*n* = 4) and NPC patients (*n* = 4). At least 400 cells have been counted for each cell line. Data are presented as mean ± SD of 3 independent experiments. **p < 0.01 in Student’s t test. **d**-**g** Co-immunofluorescence of TIA-1 and TDP-43 in NPC neuronal-like cells. Arrows indicate colocalization. Nuclei were stained with DAPi (*blue*)

Another common observation with regards to TDP-43 is its ability to colocalize with stress granules (SG). Therefore, we first evaluated whether SGs are present in cells by immunofluorescence of differentiated cells derived from healthy controls and NPC patients using the SG marker TIA-1. While in cells derived from healthy controls TIA-1 is completely localized within the nuclei, in about 64 % of NPC cells it was also detected in the cytosol (Fig. 5a, b, c). Furthermore, as shown in Fig. 5d-g, TDP-43 colocalized with TIA-1 in NPC cells indicating that in these cells cytosolic TDP-43 localized within the SG.

**Fig. 6:**
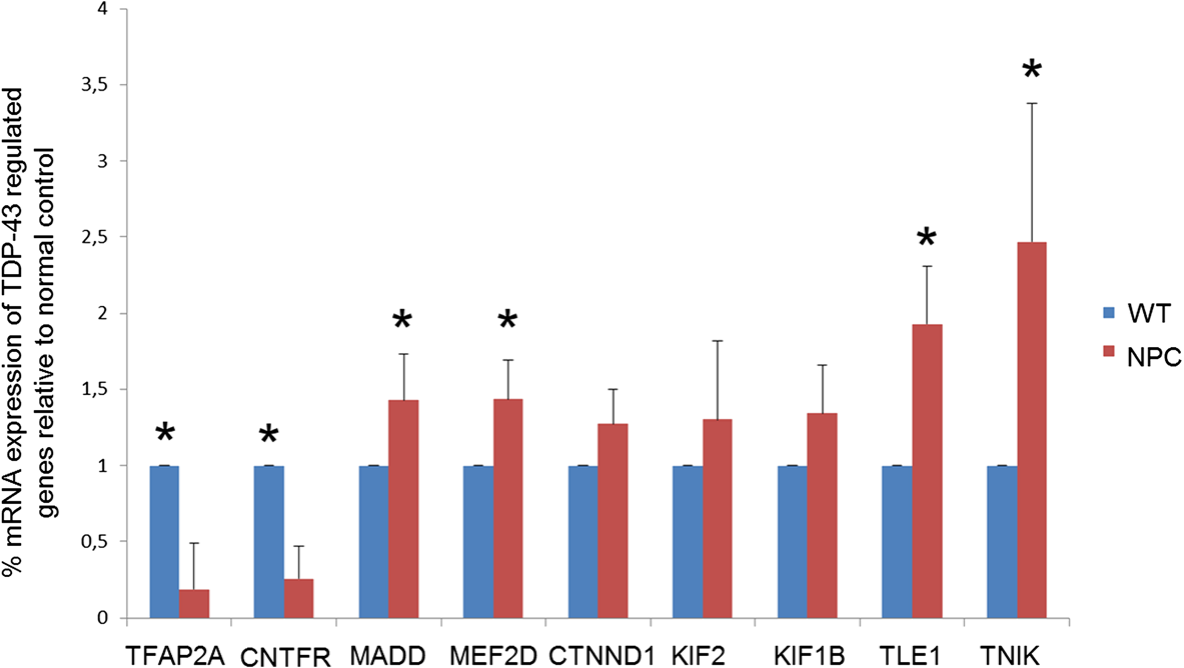
mRNA expression levels of genes regulated by TDP-43 in wt (*n* = 4) and NPC neuron-like cells (*n* = 4). TDP-43 levels were express as a percentage of wt. HPRT was used as reference gene. Data are represented as mean ± SD of at least three independent experiments. *p < 0.05

In order to investigate the possible functional impact of TDP-43 mislocalization on RNA metabolism we then analyzed the mRNA expression of a set of genes known to be regulated by TDP-43 and involved in neuronal survival and differentiation. The expression levels of nine genes were analyzed by real-time Q-PCR comparing normal vs. NPC-derived cells (Fig. 6). Importantly, we observed that six of the nine genes analyzed (*FAP2A, CNTFR, MAF2D, MADD, TLE1*, and *TNIK*) were differentially expressed in cells derived from NPC patients compared to healthy controls.

**Fig. 7:**
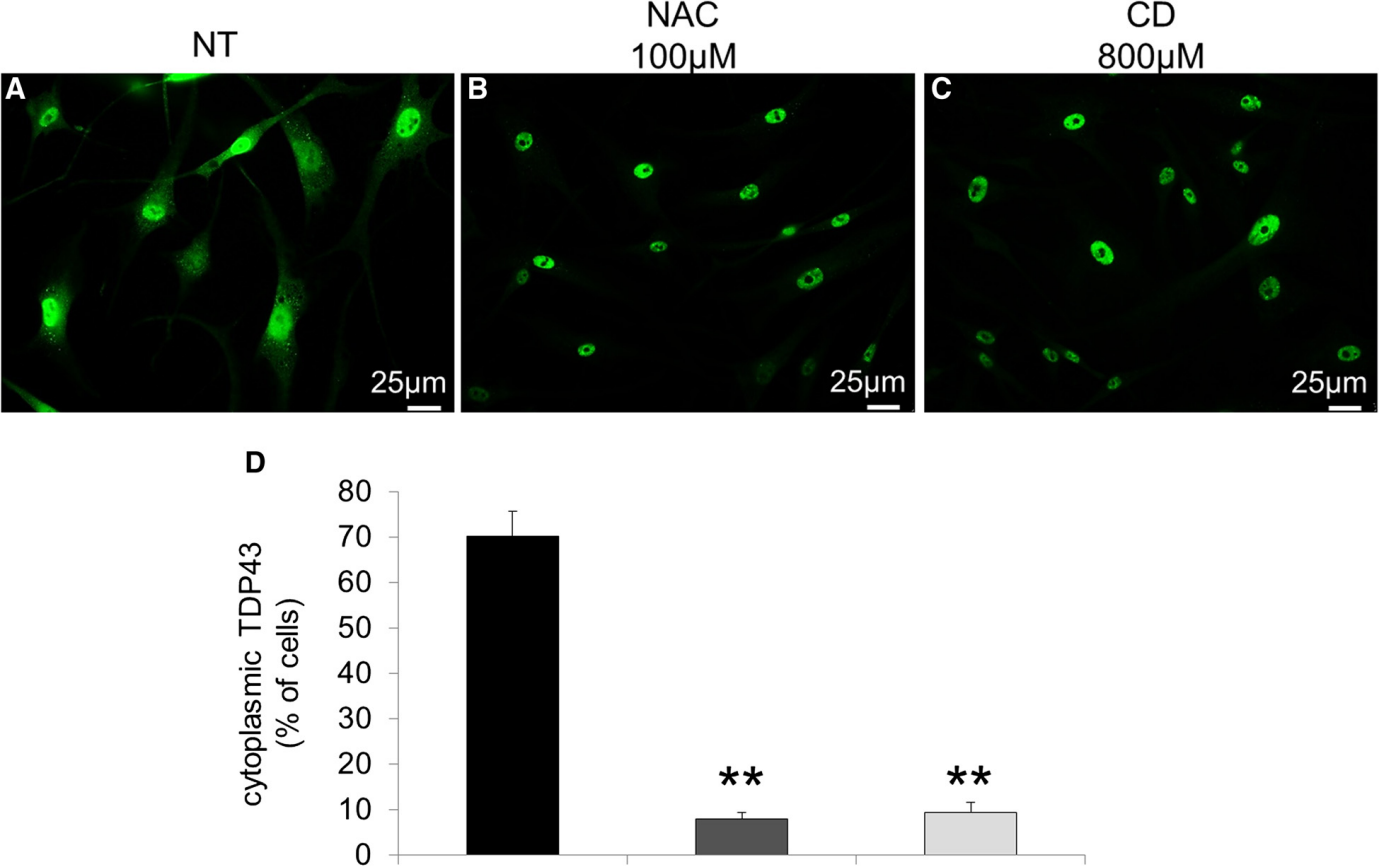
Intracellular localization of TDP-43 (labeled in *green*) in NPC neuron-like cells. Cells were incubated for 24 h with either NAC 100 μM or CD 800 μM and TDP-43 localization was examined by immunofluorescence. **a** Untreated cells (NT). **b** NAC treatment. **c** CD treatment. **d** Quantitative evaluation of the percentage of neuron like cells displaying cytoplasmic localization of TDP-43 in NPC patients (*n* = 4) untreated or treated with NAC and CD. At least 400 cells have been counted for each cell line. Data are presented as mean ± SD of 3 independent experiments. **p < 0.01 in Student’s t test. Treatment with either NAC or CD determined a reduction of TDP-43 staining in the cytosolic compartment

Since SGs are highly dynamic structures that disassemble when the stress stimuli vanish, we also analyzed whether the intracellular localization of TDP-43 could be restored by treating NPC cells with N-Acetyl-cysteine (NAC), a well known anti-oxidant agent, or with 2-hydroxypropyl-b-cyclodextrin (CD), a cholesterol sequestering agent that mobilizes cholesterol from the late endosomal/lysosomal compartment. As shown in Fig. 7, both agents almost completely restored TDP-43 localization to the nucleus.

### Analysis of TDP-43 expression/distribution in human NPC brain tissue

In order to correlate the results obtained both in the NPC mouse and in the neuronal model of the disease, the expression and localization of TDP-43 was analyzed in brain tissue obtained from a NPC patient carrying mutations c.2621A > T (D874V) and IVS14-2A > G in the *NPC1* gene (Additional file 3: Figure S2).

Atrophy was prominent in cingulate cortex and cerebellum. Neurons with swollen perikarya contained a pale granular material, a finding consistent with what is observed in storage diseases. Evidence of neuronal storage was noted in the cerebral cortex, hippocampus, amygdala, several deep telencephalic and brain stem nuclei as well as in the spinal cord. Tau-immunoreactive neurons and neurofibrillary tangles were present in hippocampus and entorhinal cortex. Tau-immunopositive threads were also noted in the spinal cord. Amyloid-β deposits were not present.

**Fig. 8:**
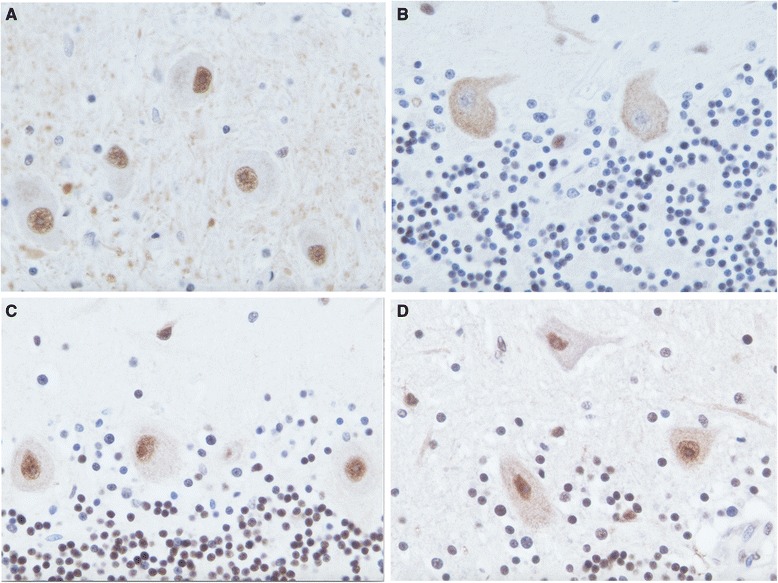
Cerebellar histologic sections immunostained with TDP43 (**a**-**d**). In the 61 year-old proband’s brain, dentate nucleus neurons show nuclear staining (**a**), while Purkinje cells show cytoplasmic staining but lack nuclear staining (**b**). In brain sections from 60 year-old (**c**) and 89 year-old (**d**) control subjects, Purkinje cells demonstrate nuclear staining with variable degrees of cytoplasmic staining. (polyclonal TDP43, 1:1000, Proteintech; original magnifications ×400)

The normal nuclear pattern of TDP-43 immunopositivity was confirmed in multiple brain areas, including the cerebellar dentate nucleus (Fig. 8a). However, in the cerebellar cortex, numerous Purkinje cells lacked nuclear TDP-43 labelling. In fact, many Purkinje cells showed diffuse TDP-43-immunopositive cytoplasmic labelling with concurrent loss of nuclear TDP43 within the same cells (Fig. 8b). In contrast, the TDP43 immunostaining of cerebellum from 4 control subjects revealed TDP43 nuclear staining in Purkinje cells with variable degrees of cytoplasmic staining, but the pattern of TDP43-negative nuclei with TDP43-positive cytoplasmic staining was not observed (Fig. 8c and d). No phospho-TDP43 positive aggregates were identified in cerebrum or cerebellum from the NPC brain (data not shown).

## Discussion

In recent years, lysosomal storage diseases have become increasingly associated with neurodegeneration and cancer [32]. Several connections between lysosomal genes and neurodegeneration have begun to emerge [33]. Lysosomal dysfunction has been suggested to play an important role in the pathogenesis of several major neurodegenerative disorders, including Parkinson disease (PD), Huntington disease (HD), and Alzheimer Disease (AD). For example, huntingtin processing occurs through the lysosomal-endosomal system leading to autophagic cell death [34], while amyloid β (Aβ) accumulation has been linked to impairment of lysosomal degradation [35]. It has been proposed that lysosomal dysfunction may also occur at an early stage in AD [36].

More recently, impaired autophagy and lysosomal dysfunction have also been linked with alterations at the level of RNA metabolism [37]. Indeed, TDP-43, an RNA binding protein implicated in neurodegeneration, has many connections with the autophagic and proteasomal system, as indicated by the requirement of multivesicular bodies for the clearance of TDP-43 aggregates within cells [38]. Therefore, we have investigated whether TDP-43, may contribute to the pathogenesis of NPC, a lysosomal storage disorder characterized by impaired intracellular lipid trafficking and autophagy.

In this work, we have shown aberrant expression and cytoplasmic distribution of TDP-43 in the cerebellum of a mouse model of NPC and in an in vitro human NPC neuronal model system in which the cytoplasmic TDP-43 is sequestered by stress granules. This TDP-43 redistribution and sequestration may represent an important link between neurodegeneration and normal biological processing, as affected by mutations, other disease processes, or prolonged environmental stress [39–41]. In addition, our findings in the human NPC neuronal model indicate that several genes reported to be under the control of TDP-43 at the RNA processing level are aberrantly expressed. This data suggests that the aberrant recruitment of TDP-43 in NPC cells might be sufficient to impair its normal function and provides a possible direct link with the neuropathology observed in NPC. However, future studies will be required to clarify these findings and better define at the molecular level the exact role of TDP-43 mislocalization in NPC pathogenesis.

In contrast to previous studies showing that p62 co-localizes with TDP-43-positive cytoplasmic inclusions in patients with FTLD-MND, we did not observe co-localization of TDP-43 and p62 in NPC cells. This finding may represent a feature specific to NPC pathology. A previous study showed that even though p62 co-localizes and physiologically interacts with TDP-43, the p62 in the cerebral cortex of FTLD-TDP patients co-immunoprecipitated a smaller amount of TDP-43 as compared with normal brains [42]. This observation suggested that the disruption of the interaction between these two proteins contributed to the pathology [42].

Besides accumulating cholesterol within the lysosomal compartment, NPC cells are characterized by oxidative stress, which may contribute to disease pathology [43, 44]. A restoration of both cholesterol trafficking and cellular oxidative status resulted in the clearance of cytosolic TDP-43 accumulation.

Based on the reported data, it is possible to hypothesize that in NPC, chronic stress conditions (including oxidative stress) may trigger the translocation of TDP-43 from the nucleus to the cytosol where it is phosphorylated and recruited within stress granules. As a consequence, its function in the regulation of RNA processing may be impaired.

The presence of TDP-43 pathology has also been observed in brain tissue obtained from a patient affected by NPC. This result is in line with the findings observed and in the human neuronal model of the disease. However, it must be interpreted with caution and confirmed by analyzing brain tissue from additional NPC patients.

Although TDP-43 pathology was observed in both *NPC−/−* mouse and in NPC human cells/tissue, the results obtained in mice do not exactly reflect the findings in human samples, suggesting that *NPC−/−* mouse would not be the best model of TDP-43 pathology in NPC. The obtained results are not surprising considering that some characteristic features of NPC human neurons, such us the high degree of ectopic dendritogenesis and the presence of neurofibrillar tangles [45, 46], are not present in mice, suggesting important specie-specific differences between mouse and human NPC neurons.

Finally, it is worth noting that in NPC cells, TDP-43 showed a diffuse cytoplasmic distribution, but no well-formed aggregates. Recently, a similar pattern of TDP-staining was described by Van Deerlin et al., in anterior horn cells and other neurons in brains from ALS patients. It was suggested that this TDP43-diffuse, often stippled or granular distribution represents a “pre-inclusion” state [47].

The reported findings are significant for several reasons. First, they potentially expand the presence of TDP-43 proteinopathies [6] beyond the ALS/FTD/AD spectrum. Second, they suggest that TDP-43 might represent a new target for the development of therapeutic interventions for NPC. In particular, it is interesting to note that beta-cyclodextrin, a compound currently under clinical evaluation for the treatment of NPC can partially restore TDP-43 nuclear localization. This observation suggests that successful drugs being developed for NPC may also find an application in the treatment of other TDP-43 proteinopathies.

## Conclusions

In conclusion, these results extend the importance of the role of TDP-43 in neurodegenerative disease and further highlight the need to prioritize the targeting of this protein to develop novel therapeutic strategies. Furthermore, our results suggest that metabolic diseases which involve neuropathological consequences could represent a novel field of study to search for alterations in RNA binding proteins, especially those that are already described to play a role in motorneuron diseases. The reason is that both the differences and similarities observed in the expression or regulation of these proteins in very different cellular disease contexts, might help us to pinpoint the basic functional alterations that trigger neurodegeneration. In addition, confirming their eventual presence in a particular metabolic disease will also represent an advantage with regards to future therapeutic strategies. In particular, it is to be expected that novel therapeutic strategies that show promise in one type of disease might also prove to be very beneficial also in other disease contexts.

## Additional files

Additional file 1: Table S1. Sequence of primers used in real-time quantitative PCR. (DOC 40 kb) Additional file 2: Figure S1. A) TDP-43 mRNA expression levels in wt and NPC neuron-like cells. HPRT has been used as reference gene. B) Western blot analysis of TDP-43 total protein levels in wt and NPC neuronal-like cells. Quantification was performed normalizing the signals of TDP-43 protein to those obtained for actin. Data are represented as mean ± SD of at least three independent experiments. No statistically significant changes were observed both in mRNA and protein TDP-43 expresion levels between NPC and healthy control cells (TIF 218 kb) Additional file 3: Figure S2. Gene figure showing 2 *NPC1* mutations, as confirmed in postmortem frozen brain tissue. (TIF 139 kb)

## References

[1] Vanier MT (2010). Niemann-Pick disease type C. Orphanet J Rare Dis.

[2] Zervas M, Somers KL, Thrall MA, Walkley SU (2001). Critical role for glycosphingolipids in Niemann–Pick disease type C. Curr Biol.

[3] Ong WY, Kumar U, Switzer RC, Sidhu A, Suresh G, Hu CY, Patel SC (2001). Neurodegeneration in Niemann-Pick type C disease mice. Exp Brain Res.

[4] Jellinger KA (2009). Recent advances in our understanding of neurodegeneration. J Neural Transm.

[5] Krecic AM, Swanson MS (1999). hnRNP complexes: composition, structure, and function. Curr Opin Cell Biol.

[6] Chen-Plotkin AS, Lee VM, Trojanowski JQ (2010). TAR DNA-binding protein 43 in neurodegenerative disease. Nat Rev Neurol.

[7] Buratti E, Baralle FE (2012). TDP-43: gumming up neurons through protein-protein and protein-RNA interactions. Trends Biochem Sci.

[8] Neumann M, Sampathu DM, Kwong LK, Truax AC, Micsenyi MC, Chou TT, Bruce J, Schuck T, Grossman M, Clark CM, McCluskey LF, Miller BL, Masliah E, Mackenzie IR, Feldman H, Feiden W, Kretzschmar HA, Trojanowski JQ, Lee VM (2006). Ubiquitinated TDP-43 in frontotemporal lobar degeneration and amyotrophic lateral sclerosis. Science.

[9] Amador-Ortiz C, Lin WL, Ahmed Z, Personett D, Davies P, Duara R, Graff-Radford NR, Hutton ML, Dickson DW (2007). TDP-43 immunoreactivity in hippocampal sclerosis and Alzheimer’s disease. Ann Neurol.

[10] Hasegawa M, Arai T, Akiyama H, Nonaka T, Mori H, Hashimoto T, Yamazaki M, Oyanagi K (2007). TDP-43 is deposited in the Guam parkinsonism-dementia complex brains. Brain.

[11] Walker AK, Daniels CM, Goldman JE, Trojanowski JQ, Lee VM, Messing A (2014). Astrocytic TDP-43 pathology in Alexander disease. J Neurosci.

[12] Wider C, Dachsel JC, Farrer MJ, Dickson DW, Tsuboi Y, Wszolek ZK (2010). Elucidating the genetics and pathology of Perry syndrome. J Neurol Sci.

[13] Buratti E, Baralle FE (2009). The molecular links between TDP-43 dysfunction and neurodegeneration. Adv Genet.

[14] Lee EB, Lee VM, Trojanowski JQ (2011). Gains or losses: molecular mechanisms of TDP43-mediated neurodegeneration. Nat Rev Neurosci.

[15] Caccamo A, Majumder S, Deng JJ, Bai Y, Thornton FB, Oddo S (2009). Rapamycin rescues TDP-43 mislocalization and the associated low molecular mass neurofilament instability. J Biol Chem.

[16] Scotter EL, Vance C, Nishimura AL, Lee YB, Chen HJ, Urwin H, Sardone V, Mitchell JC, Rogelj B, Rubinsztein DC, Shaw CE (2014). Differential roles of the ubiquitin proteasome system and autophagy in the clearance of soluble and aggregated TDP-43 species. J Cell Sci.

[17] Barmada SJ, Serio A, Arjun A, Bilican B, Daub A, Ando DM, Tsvetkov A, Pleiss M, Li X, Peisach D, Shaw C, Chandran S, Finkbeiner S (2014). Autophagy induction enhances TDP43 turnover and survival in neuronal ALS models. Nat Chem Biol.

[18] Farg MA, Sundaramoorthy V, Sultana JM, Yang S, Atkinson RA, Levina V, Halloran MA, Gleeson PA, Blair IP, Soo KY, King AE, Atkin JD (2014). C9ORF72, implicated in amytrophic lateral sclerosis and frontotemporal dementia, regulates endosomal trafficking. Hum Mol Genet.

[19] Vatovec S, Kovanda A, Rogelj B (2014). Unconventional features of C9ORF72 expanded repeat in amyotrophic lateral sclerosis and frontotemporal lobar degeneration. Neurobiol Aging.

[20] Götzl JK, Mori K, Damme M, Fellerer K, Tahirovic S, Kleinberger G, Janssens J, van der Zee J, Lang CM, Kremmer E, Martin JJ, Engelborghs S, Kretzschmar HA, Arzberger T, Van Broeckhoven C, Haass C, Capell A (2014). Common pathobiochemical hallmarks of progranulin-associated frontotemporal lobar degeneration and neuronal ceroid lipofuscinosis. Acta Neuropathol.

[21] Pacheco CD, Elrick MJ, Lieberman AP (2009). Tau deletion exacerbates the phenotype of Niemann-Pick type C mice and implicates autophagy in pathogenesis. Hum Mol Genet.

[22] Elrick MJ, Yu T, Chung C, Lieberman AP (2012). Impaired proteolysis underlies autophagic dysfunction in Niemann-Pick type C disease. Hum Mol Genet.

[23] Sarkar S, Carroll B, Buganim Y, Maetzel D, Ng AH, Cassady JP, Cohen MA, Chakraborty S, Wang H, Spooner E, Ploegh H, Gsponer J, Korolchuk VI, Jaenisch R (2013). Impaired autophagy in the lipid-storage disorder Niemann-Pick type C1 disease. Cell Rep.

[24] Elrick MJ, Lieberman AP (2013). Autophagic dysfunction in a lysosomal storage disorder due to impaired proteolysis. Autophagy.

[25] Loftus SK, Morris JA, Carstea ED, Gu JZ, Cummings C, Brown A, Ellison J, Ohno K, Rosenfeld MA, Tagle DA, Pentchev PG, Pavan WJ (1997). Murine model of Niemann–Pick C disease: mutation in a cholesterol homeostasis gene. Science.

[26] Fancello T, Dardis A, Rosano C, Tarugi P, Tappino B, Zampieri S, Pinotti E, Corsolini F, Fecarotta S, D'Amico A, Di Rocco M, Uziel G, Calandra S, Bembi B, Filocamo M. Molecular analysis of NPC1 and NPC2 gene in 34 Niemann-Pick C Italian patients: identification and structural modeling of novel mutations. Neurogenetics. 2009;10:229–39. doi:10.1007/s10048-009-0175-3. 10.1007/s10048-009-0175-3 19252935

[27] Murrell J, Farlow M, Ghetti B, Benson MD (1991). A mutation in the amyloid precursor protein associated with hereditary Alzheimer’s disease. Science.

[28] Bergamin N, Dardis A, Beltrami A, Cesselli D, Rigo S, Zampieri S, Domenis R, Bembi B, Beltrami CA (2013). A human neuronal model of Niemann Pick C disease developed from stem cells isolated from patient’s skin. Orphanet J Rare Dis.

[29] Nusca S, Canterini S, Palladino G, Bruno F, Mangia F, Erickson RP, Fiorenza MT (2014). A marked paucity of granule cells in the developing cerebellum of the *Npc1*^*−/−*^mouse is corrected by a single injection of hydroxypropyl-β-cyclodextrin. Neurobiol Dis.

[30] Canterini S, Carletti V, Nusca S, Mangia F, Fiorenza MT (2013). Multiple TSC22D4 iso-/phospho-glycoforms display idiosyncratic subcellular localizations and interacting proteinpartners. FEBS J.

[31] Takao M, Benson MD, Murrell JR, Yazaki M, Piccardo P, Unverzagt FW, Davis RL, Holohan PD, Lawrence DA, Richardson R, Farlow MR, Ghetti B (2000). Neuroserpin mutation S52R causes neuroserpin accumulation in neurons and is associated with progressive myoclonus epilepsy. J Neuropathol Exp Neurol.

[32] Ferguson SM (2015). Beyond indigestion: emerging roles for lysosome-based signaling in human disease. Curr Opin Cell Biol.

[33] Zhang L, Sheng R, Qin Z (2009). The lysosome and neurodegenerative diseases. Acta Biochim Biophys Sin (Shanghai).

[34] Martin DD, Ladha S, Ehrnhoefer DE, Hayden MR (2015). Autophagy in Huntington disease and huntingtin in autophagy. Trends Neurosci.

[35] Mueller-Steiner S, Zhou Y, Arai H, Roberson ED, Sun B, Chen J, Wang X, Yu G, Esposito L, Mucke L, Gan L (2006). Antiamyloidogenic and neuroprotective functions of cathepsin B: implications for Alzheimer’s disease. Neuron.

[36] Nixon RA, Cataldo AM, Mathews PM (2000). The endosomal-lysosomal system of neurons in Alzheimer’s disease pathogenesis: a review. Neurochem Res.

[37] Buratti E (2015). Functional Significance of TDP-43 Mutations in Disease. Adv Genet.

[38] Filimonenko M, Stuffers S, Raiborg C, Yamamoto A, Malerød L, Fisher EM, Isaacs A, Brech A, Stenmark H, Simonsen A (2007). Functional multivesicular bodies are required for autophagic clearance of protein aggregates associated with neurodegenerative disease. J Cell Biol.

[39] Wolozin B (2012). Regulated protein aggregation: stress granules and neurodegeneration. Mol Neurodegener.

[40] Bentmann E, Haass C, Dormann D (2013). Stress granules in neurodegeneration--lessons learnt from TAR DNA binding protein of 43 kDa and fused in sarcoma. FEBS J.

[41] Li YR, King OD, Shorter J, Gitler AD (2013). Stress granules as crucibles of ALS pathogenesis. J Cell Biol.

[42] Tanji K, Zhang HX, Mori F, Kakita A, Takahashi H, Wakabayashi K (2012). p62/sequestosome 1 binds to TDP-43 in brains with frontotemporal lobar degeneration with TDP-43 inclusions. J Neurosci Res.

[43] Zampieri S, Mellon SH, Butters TD, Nevyjel M, Covey DF, Bembi B, Dardis A (2009). Oxidative stress in NPC1 deficient cells: protective effect of allopregnanolone. J Cell Mol Med.

[44] Fu R, Yanjanin NM, Bianconi S, Pavan WJ, Porter FD (2010). Oxidative stress in Niemann-Pick disease, type C. Mol Genet Metab.

[45] Love S, Bridges LR, Case CP (1995). Neurofibrillary tangles in Niemann-Pick disease type C. Brain.

[46] Walkley SU, Suzuki K (2004). Consequences of NPC1 and NPC2 loss of function in mammalian neurons. Biochim Biophys Acta.

[47] Van Deerlin VM, Leverenz JB, Bekris LM, Bird TD, Yuan W, Elman LB, Clay D, Wood EM, Chen-Plotkin AS, Martinez-Lage M, Steinbart E, McCluskey L, Grossman M, Neumann M, Wu I-L, Yang W-S, Kalb R, Galasko DR, Montine TJ, Trojanowski JQ, Lee VM-Y, Schellenberg GD, Yu C-E (2008). TARDBP mutations in amyotrophic lateral sclerosis with TDP-43 neuropathology: a genetic and histopathological analysis. Lancet Neurol.

