# Supplementary material for: Altered localization and functionality of TAR DNA Binding Protein 43 (TDP-43) in niemann- pick disease type C
Source: Acta Neuropathol Commun. 2016 May 18;4:52. doi: 10.1186/s40478-016-0325-4 (PMC4870731; doi:10.1186/s40478-016-0325-4)
Supplement: Additional file 1: Table S1. — Sequence of primers used in real-time quantitative PCR. (DOC 40 kb) [file 40478_2016_325_MOESM1_ESM.doc]

SUPPLEMENTARY TABLE 1: sequence of primers used in real-time quantitative PCR.

| **Gene** | **Primer sequence** |
| --- | --- |
| CNTFR | Forward: 5'-GCGACTCTAGCCTTGTCACC-3' |
|  | Reverse: 5'-ACAGGAGCAGCCATCTCTTC-3' |
| CTNND1 | Forward: 5'-AAGGAGGAAGCAGGAAGGAG-3' |
|  | Reverse: 5'-TTGAGGTGTGTGGCTTTTGA-3' |
| HPRT | Forward: 5'-GACCAGTCAACAGGGGACA-3' |
|  | Reverse: 5'-GTGTCAATTATATCTTCCACAATCAAG-3' |
| KIF1B | Forward: 5'-TCGAAGCTGAAGGACTTTGG-3' |
|  | Reverse: 5'-TCGAGAGACCAGCAAGGAAT-3' |
| KIF2A | Forward: 5'-ATTGGACGAACATCACCAGC-3' |
|  | Reverse: 5'-AGGAATGGCATCCTGTGAAA-3' |
| MADD | Forward: 5'-AGACTCGCTGGCTCACATCT-3' |
|  | Reverse: 5'-TTGAGACCAACTCTGCCACA-3' |
| MEF2D | Forward: 5'-CTCATGAACGGTCTGGGAAC-3' |
|  | Reverse: 5'-CTCTTTGCCGTGACAACACC-3' |
| TFAP2A | Forward: 5'-TCCAACAGCAATGCCGTCTC-3' |
|  | Reverse: 5'-GCCACCGTGACCTTGTACTT-3' |
| TLE1 | Forward: 5'-CGTATTCAATCTCTTGGCGA-3' |
|  | Reverse: 5'-TGGAATGTGAGAAACTGGCA-3' |
| TNIK | Forward: 5'-TCCTCTTCATCCCCTGTGAC-3' |
|  | Reverse: 5'-TGGAACATACGGGCAAGTTT-3' |
| TIA1 | Forward: 5'-CATGGAACCAGCAAGGATTT-3' |
|  | Reverse: 5'-CACTCCCTGTAGCCTCAAGC-3' |
| HPRT | Forward: 5'-GACCAGTCAACAGGGGACAT -3' |
|  | Reverse: 5'-GTGTCAATTATATCTTCCACAATCAAG-3' |
