# Supplementary figures and images for: Altered localization and functionality of TAR DNA Binding Protein 43 (TDP-43) in niemann- pick disease type C
Source: Acta Neuropathol Commun. 2016 May 18;4:52. doi: 10.1186/s40478-016-0325-4 (PMC4870731; doi:10.1186/s40478-016-0325-4)

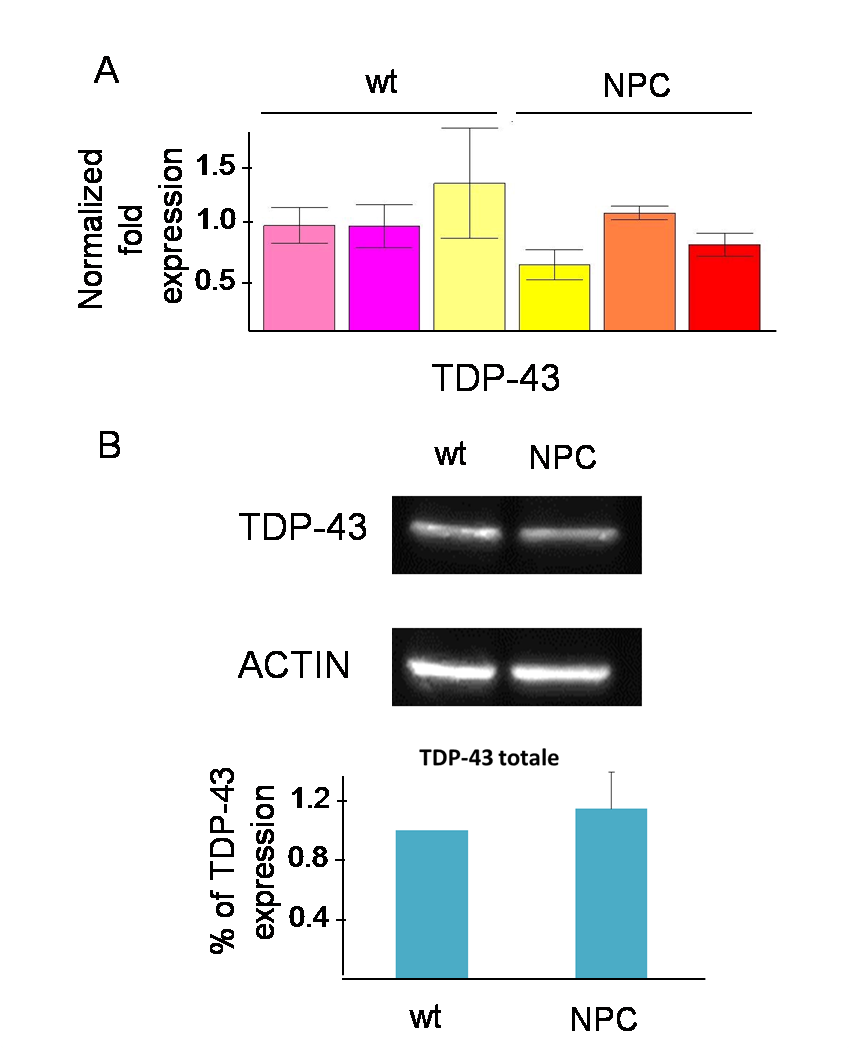

Supplement: Additional file 2: Figure S1. — A) TDP-43 mRNA expression levels in wt and NPC neuron-like cells. HPRT has been used as reference gene. B) Western blot analysis of TDP-43 total protein levels in wt and NPC neuronal-like cells. Quantification was performed normalizing the signals of TDP-43 protein to those obtained for actin. Data are represented as mean ± SD of at least three independent experiments. No statistically significant changes were observed both in mRNA and protein TDP-43 expresion levels between NPC and healthy control cells (TIF 218 kb) [file 40478_2016_325_MOESM2_ESM.tif]

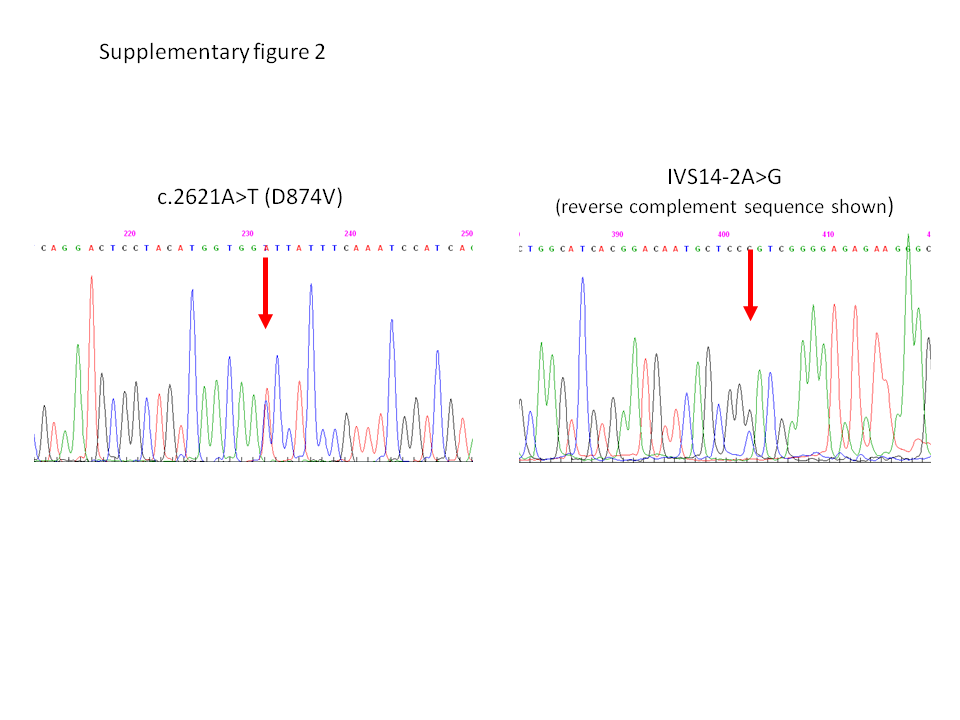

Supplement: Additional file 3: Figure S2. — Gene figure showing 2 NPC1 mutations, as confirmed in postmortem frozen brain tissue. (TIF 139 kb) [file 40478_2016_325_MOESM3_ESM.tif]
